# *In situ* visualisation of the abundant Chloroflexi populations in full-scale anaerobic digesters and the fate of immigrating species

**DOI:** 10.1371/journal.pone.0206255

**Published:** 2018-11-01

**Authors:** Francesca Petriglieri, Marta Nierychlo, Per Halkjær Nielsen, Simon Jon McIlroy

**Affiliations:** Center for Microbial Communities, Department of Chemistry and Bioscience, Aalborg University, Aalborg, Denmark; University of Notre Dame, UNITED STATES

## Abstract

Anaerobic digestion is a key process for the conversion of waste organics to biogas for energy and is reliant on the synergistic activities of complex microbial communities. Members of the phylum Chloroflexi are often found to be abundant in these systems, yet little is known of their role, with most members yet to be cultured or identified. The aim of this study was to characterize the Chloroflexi communities present in full-scale anaerobic digesters receiving excess sludge from wastewater treatment plants. The core genus-level-phylotypes were identified from extensive 16S rRNA gene amplicon sequencing surveys of 19 full-scale systems over a 6 year period. The T78 and *Leptolinea*, and the RB349 and SJA-170, were found to be the most abundant genera of mesophilic and thermophilic digesters, respectively. With the exception of *Leptolinea*, these phylotypes are known only by their 16S rRNA gene sequence, and their morphology and metabolic potentials are not known. Fluorescence *in situ* hybridisation (FISH) probes were designed for these phylotypes, with their application revealing a similar thin filamentous morphology, indicating a possible role for these organisms in maintaining floc structure. The new FISH probes provide a useful tool for future efforts to characterize these organisms *in situ*. FISH also suggests that immigrating Chloroflexi species die off in the anaerobic digester environment and their high abundance in anaerobic digesters, observed with DNA based sequencing surveys, was quite possibly due to the persistence of their DNA after their death. This observation is important for the interpretation of popular DNA-based sequencing methods applied for the characterisation of communities with substantial immigration rates, such as anaerobic digesters.

## Introduction

Anaerobic digestion (AD) is a complex biochemical process that is widely applied in wastewater treatment for sludge reduction and the recovery of valuable resources. The system is strictly dependent on the tightly coupled synergistic interactions between the microorganisms of complex communities, which are able to convert organic matter to methane for energy, and consists of four main microbial-mediated steps: hydrolysis, fermentation, acetogenesis and methanogenesis [1–3]. The optimal design and operation of anaerobic digester systems will likely require an in depth understanding of the organisms responsible. Even though the basic overall transformations of AD have been well studied, little is known about the microbial community structure and dynamics in full-scale digesters, and most of the key microorganisms remain to be cultured or further identified and characterized [4].

Surveys of the bacterial community in full-scale ADs, based on 16S rRNA gene sequencing, have often reported the phylum Chloroflexi as one of the most abundant, suggesting a substantial contribution and importance of these to the overall transformations that underpin the function of AD systems [1,4,5]. Moreover, preliminary evidence suggests a potential role for members of the phylum in the operational problem of foaming in AD systems (Jiang, C., Nielsen P.H., and others, unpublished). Despite their likely importance, very little is known of the metabolic activity or potential of Chloroflexi populations in ADs. Large scale DNA-based surveys reveal that most of the AD-related Chloroflexi species are not classified beyond the class or family [1,4,5], and inference of metabolic traits becomes unreliable for classification at taxonomic levels higher than the species or genus [6]. In order to address these issues, the MiDAS initiative has sought to assign genus level phylogenetic annotation to all abundant members of the AD community, to which information on the distribution, morphology and metabolic activities of members of these genera can be accumulated [7]. Based on a 16S rRNA gene amplicon survey of 19 full-scale Danish AD plants over a 6-year period [4], almost all of the abundant genus-level-phylotypes within the Chloroflexi were novel and given unique identifiers in the MiDAS taxonomy [7]. Characterisation of the abundant core genera is key to understanding the role of the Chloroflexi in AD systems.

A key complementary method to the high-throughput 16S rRNA amplicon sequencing surveys [4] is fluorescence *in situ* hybridisation (FISH). An advantage of FISH is that it is not subject to the same DNA extraction and amplification biases as sequencing-based methods and can therefore be used to verify the abundance of key groups of microorganisms [8]. The method also allows the *in situ* visualization of the morphology and spatial arrangement of cells [9], potentially giving key insights into their ecology—such as the potential synergistic relationship between *Methanosaeta* methanogens and the fermentative “Brevefilum spp.” observed to be co-located in AD systems [10]. In addition, when coupled with methods like microautoradiography (MAR) and histochemical staining, key metabolic traits can be directly confirmed *in situ* [11].

When considering the microbial community of anaerobic digesters, it is important to recognize that a large portion of the population is immigrating with the activated sludge biomass fed into the system [4,12]. Immigrating activated sludge populations are routinely observed in high abundance in recipient AD systems with DNA-based methods, yet it is unclear whether they are active members of the AD community or if their naked DNA is simply persisting in the system [4]. *In situ* characterisation studies of Chloroflexi abundant in activated sludge has suggested they are fermentative facultative anaerobes and are therefore potentially able to survive in the AD environment [13,14]. Even if the organisms are inactive, persistence of intact hydrophobic cells could stabilize foam in the AD system [15]. This could well be the case for members of the ‘*Candidatus* Defluviifilum’, which are abundant members of the activated sludge community shown to have a hydrophobic surface [13]. Application of FISH could be used to give an insight into these important questions.

The overall aim of this study was to characterize the Chloroflexi community in full-scale AD systems. The large scale MiDAS survey data [7] was analysed to identify the genus level taxa consistently found to be abundant across these plants. FISH probes were designed for their *in situ* visualisation and provide a resource for future characterisation of their ecophysiology. Quantitative FISH was also applied to investigate the fate of immigrating filamentous Chloroflexi populations abundant in the activated sludge biomass fed into the digesters.

## Materials and methods

### Identification of target organisms

An initial screening of microbial communities of full-scale anaerobic digesters was realized using the MiDAS database [7], covering activated sludge from 15 WWTPs, mesophilic anaerobic digesters from 26 reactors at 14 WWTPs, and thermophilic ADs from 7 reactors at 5 WWTPs, sampled 2–4 times a year over a period of 6 years (2011–2016) (see [4] for plant operation details). Sampling, DNA extraction, 16S rRNA amplicon sequencing, and data processing were performed as described by Stokholm-Bjerregaard et al., [16]. Briefly, after sample collection, DNA was extracted using the FastDNA spin kit for soil (MP Biomedicals), following the manufacturer’s protocol, except for an increase of the bead beating to 4 x 40 s at 6 m/s using a FastPrep FP120 (MP Biomedicals). Approximately 10 ng of extracted DNA was then used as template for PCR amplification, using V1-3 primers (27F AGAGTTTGATCCTGGCTCAG and 534R ATTACCGCGGCTGCTGG) [17]. The reaction mixture also contained a pair of barcoded library adaptors (400 nM), MgSO_4_ (1.5 mM), dNTPs (400 nM of each), Platinum Taq DNA polymerase high fidelity (HF) (2 mU) and 1 x Platinum High Fidelity buffer (Thermo Fisher Scientific). The reaction settings consisted of an initial denaturation at 95°C for 2 min, 30 cycles of 95°C for 20 s, 56°C for 30 s, 72°C for 60 s, and final elongation at 72°C for 5 min. The amplicon libraries, generated in duplicates and pooled together, were then purified using the Agencourt AMpure XP bead protocol (Beckmann Coulter, Brea, CA, USA) with a sample/bead solution ratio of 5/4 and the DNA elution in 33 mL nuclease-free water. DNA concentration was measured with Quant-iTTM HS DNA Assay (Thermo Fisher Scientific) and libraries’ quality was validated with a Tapestation 2200 using D1K ScreenTapes (Agilent). The libraries were then pooled in equimolar concentrations, diluted to 4 nM and sequenced on a MiSeq (Illumina) following essentially the procedure in Caporaso et al., [18]. After removing low quality sequences from the raw data, forward and reverse reads were merged together and screened for potential chimeric sequences. The merged reads were then clustered into OTUs with 97% similarity, and taxonomic classification was performed using MiDAS version 2.1 [7]. The datasets obtained were analysed using R (R Core Team, 2014), through the Rstudio IDE3 and visualized using the ampvis package [8].

### Biomass sampling and fixation

Biomass samples from full-scale anaerobic digesters were fixed with cold 4% [w/v] paraformaldehyde (final concentration) for 3 h at 4°C. After centrifugation (8 min at 4500 rpm), the supernatant was removed and the samples were washed 3 times with sterile filtered tap water and resuspended in 50% [v/v] ethanol in 1 × phosphate buffered saline (PBS). The fixed samples were stored in the freezer (-20°C) until needed.

### FISH

FISH was performed as described by Daims et al., [19]. Unless otherwise stated, a 3 h hybridisation period was determined to be sufficient for optimal fluorescent signal for the target group. Details about the optimal formamide concentration used for each probe are given in Table 1. The nonsense NON-EUB probe was applied to all samples as a negative control for sequence independent probe binding [20]. As a general stain for all microorganisms, 3.6 μM 4',6-diamidino-2-phenylindole (DAPI) was applied for 1 h at 4°C in the dark. Quantitative FISH estimations of target populations was performed using the DAIME software [21]. Biovolumes were calculated from 30 images acquired with a 63 x magnification objective lens. The abundance was measured as the percentage of the area fluorescing with the EUBmix [22,23] and ARC915 [24] (both Cy5 labeled, collectively covering most Bacteria and Archaea) that also fluoresced with the target probe (Cy3). Microscopic analysis was performed with an Axioskop epifluorescence microscope (Carl Zeiss, Germany), equipped with a LEICA DFC7000 T CCD camera or a white light laser confocal microscope (Leica TCS SP8 X) (Leica Microsystems, Kista, Sweden).

**Table 1:** Probes designed and optimized in this study.

Probe	*E*. *coli* pos.	Target group	Coverage*	Non-target hits	Sequence (5’-3’)	[FA]%**
**CFX593**	**593–615**	**T78**	**605/663**	**2**	**GGA GCT TTC ACG CCC TAC TTA C**	**35**
CFX593_H1	616–637	Helper for CFX593	N/A	N/A	ACC ACC TRC ACG CGC TTT ACG C	N/A
CFX593_H2	572–592	Helper for CFX593	N/A	N/A	CGD CMT CTC CCA GTT GAG CC	N/A
CFX593_C1	593–615	Competitor for CFX593 probe	N/A	N/A	GGA GCT TTC ACG CCC AAC TTA C	N/A
**CFX357**	**357–379**	**T78**	**28/663**	**0**	**GCC CAT TTG CAA TAT TCC CTA C**	**35**
CFX357_H1	380–402	Helper for CFX357	N/A	N/A	TGC TGC CAC CCG TAG GTG TAT G	N/A
CFX357_H2	332–356	Helper for CFX357	N/A	N/A	GCG GCG TTG CTA CAT CAG GCT TTC	N/A
**CFX790**	**790–810**	***Leptolinea***	**65/105**	**9**	**GCT AAG ACT ACC GGG GTC TCT**	**40**
CFX790_H1	768–789	Helper for CFX790	Nagt/A	N/A	AAT CCC GTT TGC TAC CYT AGC T	N/A
CFX790_H2	811–836	Helper for CFX790	N/A	N/A	CCG ACR CYA AGT TCA CAT CGT TTA CA	N/A
CFX790_C1	790–810	Competitor for CFX790 probe	N/A	N/A	GCT AAG ACT ACC GGG GTA TCT	N/A
CFX790_C2	790–810	Competitor for CFX790 probe	N/A	N/A	GCT AGG ACT ACC GGG GTC TCT	N/A
CFX790_C3	790–810	Competitor for CFX790 probe	N/A	N/A	GCT AAG ACT ACR GGG GTC TCT	N/A
**CFX837**	**837–857**	***Leptolinea***	**41/105**	**3**	**AGT ACC GAT GGA TTT AAC CCC A**	**30**
CFX837_H1	808–836	Helper for CFX837	N/A	N/A	CCG ACA CTA AGT TCA CAT CGT TTA CAG CT	N/A
CFX837_H2	860–885	Helper for CFX837	N/A	N/A	CAG GCG GTG AAC TTA TCG CGT TWG CT	N/A
**CFX626**	**626–644**	**SJA-170**	**3/14**	**0**	**AGT TTT GAC CGA CCT CYC C**	**30**
CFX626_H1	598–619	Helper for CFX626 probe	N/A	N/A	AGC CRG GRG CTT TCA CAG CCA A	N/A
CFX626_H2^#^	663–684	Helper for CFX626 probe	N/A	N/A	ACC CGG AAT TCC ATC TYC CTC T	N/A
CFX626_C1	626–644	Competitor for CFX626	N/A	N/A	AGT TTT GAC TGA CCT CTC C	N/A
CFX626_C2	626–644	Competitor for CFX626	N/A	N/A	AGT TTT GTC CGA CCT CTC C	N/A
CFX626_C3^¤^	626–644	Competitor for CFX626	N/A	N/A	AGT TTT GAR CGA CCT CYC C	N/A
**CFX428**	**428–448**	**RB349**	**25/38**	**0**	**TCA GCC AGA AAA GCC CTT TAC**	**25**
CFX428_H1	420–397	Helper for CFX428 probe	N/A	N/A	AGG CCK TCN TCR DGC ACG CGG CGT	N/A
CFX428_H2^#^	481–503	Helper for CFX428 probe	N/A	N/A	ACD CTT ATT CCT GGC YTA CHG TCC	N/A
CFX428_C1	428–448	Competitor for CFX428 probe	N/A	N/A	TCC TCC AGA AAA GCC CTT TAC	N/A
**CFX745**	**745–763**	**RB349**	**22/38**	**0**	**CGG CTC AGC GTC AGG TGC A**	**40**
CFX745_H1^#^	713–733	Helper for CFX745 probe	N/A	N/A	CGC CTT CGC CTC TGG TGT TCC	N/A
CFX745_H2^#^	778–800	Helper for CFX745 probe	N/A	N/A	CCG GGG TTT CTA ATC CCG TTC GC	N/A
CFX745_C1	745–763	Competitor for CFX745 probe	N/A	N/A	CGH CTC AGC GTC AGG TGC A	N/A
CFX745_C2	745–763	Competitor for CFX745 probe	N/A	N/A	CAT CTC AGC GTC AGG TGC A	N/A

* Taxonomy and coverage of groups is defined as in the MiDAS database (Release 2.1) [7].

Values given as group hits/ group totals; N/A = Not applicable;

**Recommended optimal formamide concentration for use in FISH hybridisations.

^#^ Helper probes not required for optimal fluorescent signal of their respective probe. If an isolate with matched target site was available, competitor probes were assessed against it. If the mismatch alone is enough to prevent non-target binding of the labeled probe, at the recommended formamide concentration, then the competitor probe is indicated as not required (¤) (note: it is recommended that competitor probes are included for un-validated mismatches).

### Phylogenetic analysis and FISH probe design

Phylogenetic analysis of 16S rRNA gene sequences and design of FISH probes for the target organisms were performed using the ARB software [25]. A phylogenetic tree was calculated based on the aligned 16S rRNA gene nucleotide sequences, using the maximum likelihood method and a 1000-replicate bootstrap analysis. Unlabeled helper probes and competitor probes were designed for predicted inaccessible regions and for single base mismatched non-target sequences, respectively. Potential probes were validated *in silico* with the MathFISH software for hybridization efficiencies of target and potentially weak non-target matches [26]. The existence of non-target indel sequences was checked with the Ribosomal Database Project (RDP) “PROBE MATCH” function [27,28]. All probes were purchased from Sigma-Aldrich (Denmark), labeled with 5(6)-carboxyfluorescein-N-hydroxysuccinimide ester (FLUOS), indocarbocyanine (Cy3) or indodicarbocyanine (Cy5) fluorochromes. Optimal hybridization conditions for novel FISH probes were determined based on formamide dissociation curves, generated after hybridization at different formamide concentrations, over a range of 15–70% (v/v) with 5% increments. Relative fluorescent intensities of 50 cells were measured with ImageJ software (National Institutes of Health, Maryland, USA) and calculated average values were compared for selection of the optimal formamide concentration. Where available, pure cultures were obtained from DSMZ and applied in the optimization process. *Leptolinea tardivitalis* (DSM16556) was used to optimize the probe CFX790, while *Ornatilinea apprima* (DSM23815) and *Geobacillus stearothermophilus* (DSM22) were used to assess the need of the specific unlabeled competitor probes CFX790_C2, CFX626_C3 and CFX745_C1, respectively. If pure cultures were not available, probes were optimized using AD biomass with a high abundance of the target organism predicted by amplicon sequencing.

## Results and discussion

### Distribution of Chloroflexi in full-scale anaerobic digesters

**Fig 1:**
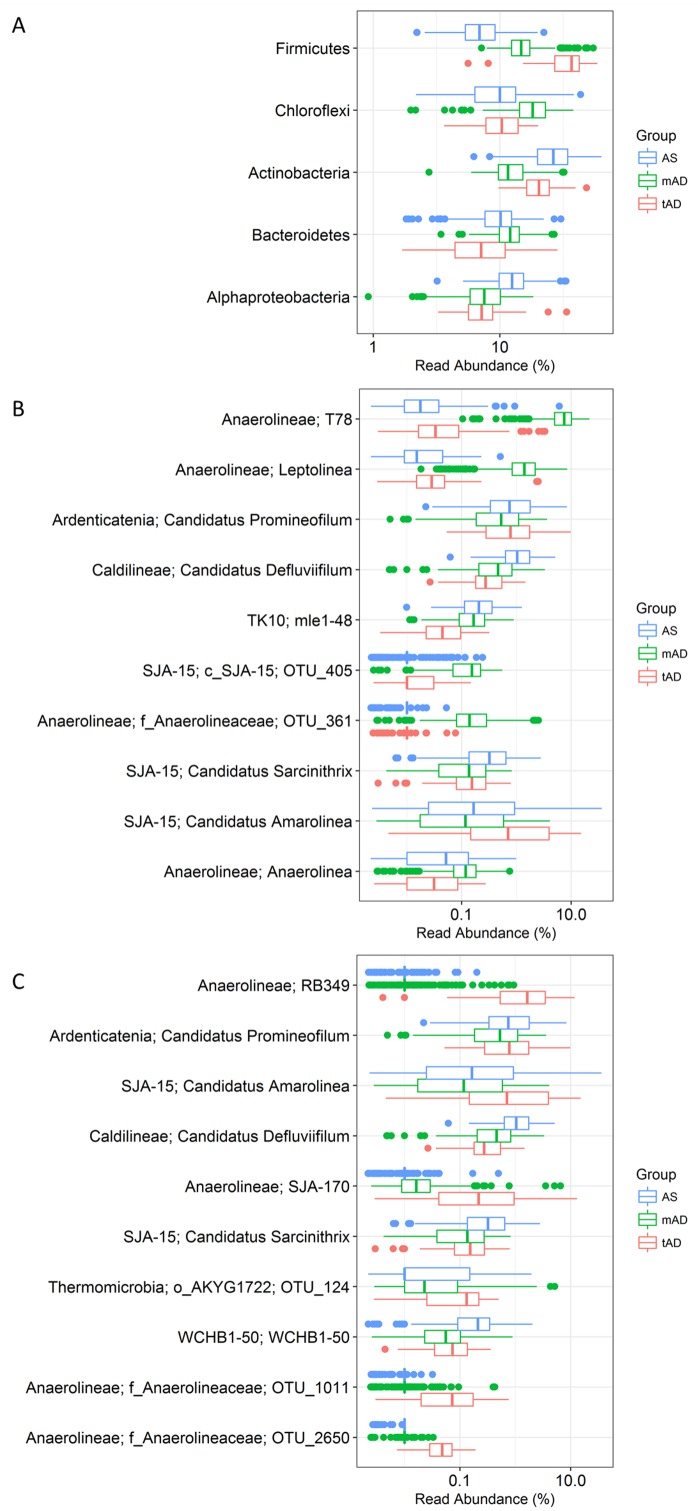
Distribution of Chloroflexi in full-scale anaerobic digesters and in activated sludge fed therein. **A**. 5 most abundant phyla in Danish AS and AD. **B.** 10 most abundant Chloroflexi genera in mesophilic ADs (mAD) and their corresponding abundance in thermophilic ADs (tAD) and AS. **C.** 10 most abundant Chloroflexi genera in thermophilic ADs and their corresponding abundance in mesophilic AD and AS. X-axis shows the relative read abundance in percentage of total bacteria.

Analysis of the MiDAS survey was performed to give an overview of genus-level-taxa belonging to the phylum Chloroflexi in anaerobic digesters and the activated sludge feed. In anaerobic digesters (Fig 1A), the most abundant phyla were Firmicutes, Chloroflexi, Bacteroidetes, Actinobacteria, and Proteobacteria. The high abundance of Chloroflexi in the anaerobic digesters investigated is in agreement with other sequence-based studies of the microbial community of ADs [1,4,5].

Among the Chloroflexi populations abundant in the AD, a few genera were apparently specialized to either mesophilic or thermophilic conditions. In mesophilic systems, the T78 were the most abundant of all the bacterial genera, and members of the genus *Leptolinea* were also found in high abundance. The results were similar in thermophilic ADs (Fig 1C), which showed high abundance of Chloroflexi genera, such as RB349 and SJA-170. All of these genera are classified within the family Anaerolineaceae. This observation is consistent with earlier DNA based surveys which found Chloroflexi in AD systems were almost exclusively within this family [1,5]. The noticeably lower abundance of these genera in the feed for these systems (activated sludge) strongly suggests that they are growing in the AD environment. Studies of AD isolates [29–32] and metagenomically derived genomes of uncultured AD species [10,33] classified to the Anaerolineaceae family, along with *in situ* studies of the phylum Chloroflexi [34], suggest a general likely role in fermentation of carbohydrates and proteinaceous material. So far, *Leptolinea tardivitalis* [30] and the uncultured “Brevefilum fermentans” (formerly the A6 MiDAS phylotype) [10], represent the only members of abundant Chloroflexi genera in full-scale systems to be partially characterized.

**Fig 2:**
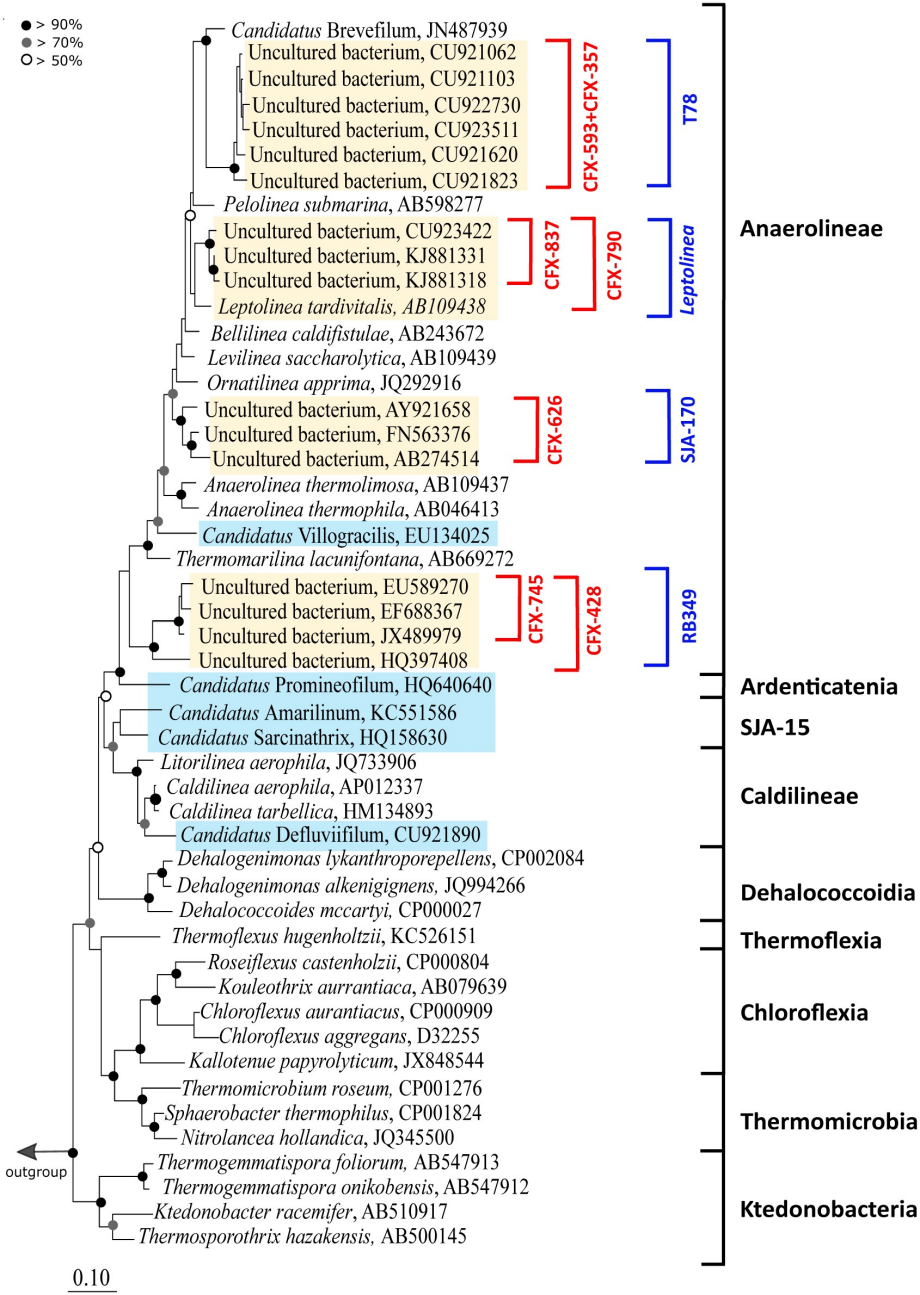
Maximum likelihood (PhyML) 16S rRNA gene phylogenetic tree of abundant members of the Chloroflexi in anaerobic digesters. A 20% conservational filter was applied to the alignment used for the tree to remove hypervariable regions, giving 1125 positions. Phylogenetic classification is based on the MiDAS database (Release 2.1) and is indicated with black brackets. Note that in this MiDAS release *Candidatus* Sarcinithrix, *Candidatus* Villigracilis and *Candidatus* Amarolinea are designated as *Candidatus* Sarcinathrix, *Candidatus* Villogracilis and *Candidatus* Amarilinum, respectively. The corrected names [35] appear in this phylogenetic tree, are used in this manuscript and will be updated in subsequent MiDAS releases. Probe coverage is shown with red brackets. Phylotypes abundant in AD and AS are highlighted in yellow and cyan, respectively. Bootstrap values from 1000 re-samplings are indicated for each branch. Species of the phylum Cyanobacteria were used as the outgroup. The scale bar represents substitutions per nucleotide base.

FISH probes were designed to visualize the morphology and spatial arrangement of the phylotypes abundant in both types of AD. An overview of the phylogeny of Chloroflexi phylum and the coverage of the novel FISH probes designed is shown in Fig 2. In general, it was difficult to design FISH probes to cover all sequences belonging to the individual groups (Table 1). As such, probes were designed to at least target sequences closely related to the abundant amplicon sequences of the MiDAS survey to ensure the abundant members of AD communities were covered.

**Fig 3:**
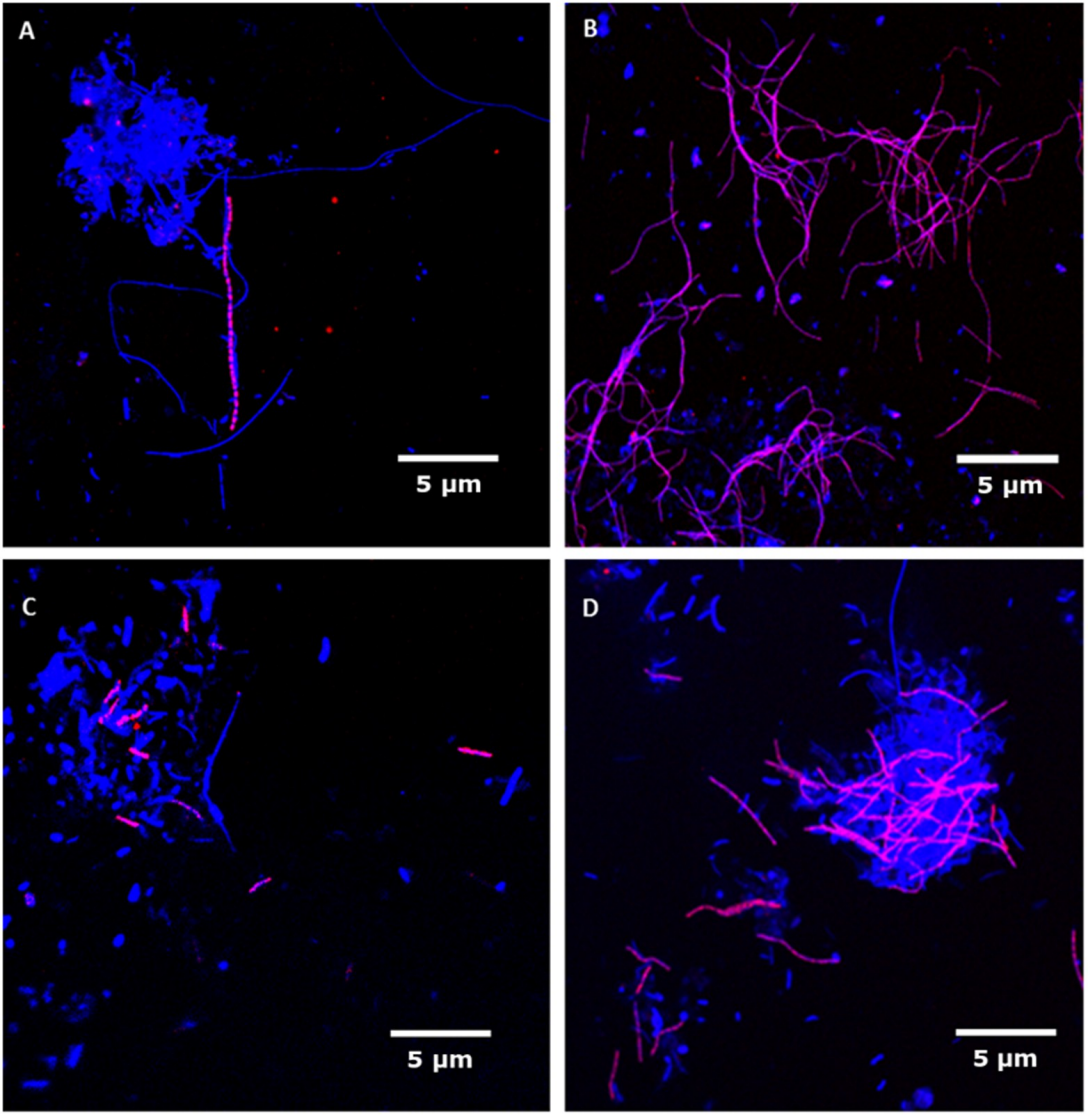
Composite FISH micrographs of Chloroflexi genera in full-scale anaerobic digesters. The specific probes (Cy3-label, red) target (**A**) T78, (**B**) Leptolinea, (**C**) SJA-170 and (**D**) RB349, while DAPI staining (blue) is used to show all microbial cells. Target filaments appear magenta, while all other cells are blue. Scale bars represent 5 μm.

Since a candidate for a single probe to cover the mesophilic T78 phylotype was not identified, the CFX357 and CFX593 were designed. These two probes collectively target most of the genus with high coverage and specificity. Helper probes were required for an optimal FISH signal (Table 1). Application of these probes revealed members of the genus to be filamentous, between 5 and 80 μm long and 0.6 μm thick, unbranched, and were often found in bundles or protruding from the surface of flocs (Fig 3A). No epiphytic cell growth attached to the filaments was observed. The CFX790 and CFX837 probes were designed to cover the mesophilic genus *Leptolinea* with reasonable coverage. The CFX790 probe has broader coverage of the genus but lower specificity than CFX837 (Table 1). Applying both probes together with different fluorochromes, and assessing their overlap is recommended to give the highest confidence in specificity. Both probes required an overnight hybridisation and the use of helper probes to detect a fluorescent signal. The *Leptolinea* formed filaments, 0.5 μm thick and up to 25 μm long but typically less than 5 μm in length (Fig 3B). No filament branching or attached growth were observed. In contrast to the observed length of *Leptolinea* spp. in this study, the pure culture of the isolate *Leptolinea tardivitalis* has been shown to form filaments longer than 100 μm [30]. The *Leptolinea* spp. were distributed throughout the AD flocs, but mainly concentrated near the floc surface.

For the Chloroflexi abundant in thermophilic AD, the CFX626 probe was designed to detect the SJA-170 phylotype and the CFX428 and CFX745 probes to detect the RB349 phylotype. Helper probes were necessary to produce a good fluorescent signal for CFX626 and CFX428, but were not essential for CFX745. The cell morphology of SJA-170 and RB349 appeared very similar (Fig 3C and 3D), with short filaments up to 20 μm long, approximately 0.5 μm thick, without branching or attached growth. They were typically present inside the flocs but were also observed to protrude from them or to be dispersed in the mixed liquor.

Given the similar morphology of these four abundant AD phylotypes, it was tested and confirmed that the probes were not overlapping in coverage. This was achieved by applying each probe set to samples where sequencing had confirmed that non-target groups were relatively high in abundance, and the target group was very low in abundance or not detected, and confirming that few to no filaments gave a positive FISH signal. The relatively high abundance of other groups in the sample was also confirmed with the separate application of their respective FISH probes. The filamentous morphology of these abundant AD phylotypes could indicate a role in maintenance of floc structure. Filamentous members of the phylum were suggested to be important structural components of granules in upflow anaerobic sludge blanket reactors [36] and also in activated sludge flocs [37]. However, in both these previous studies the Chloroflexi filaments were longer (15–140 μm) than those observed for the AD phylotypes analysed in this study (all < 80 μm in length). No noticeable physical association of these phylotypes with the methanogens present, as was reported for the “Brevefilum spp.” [10], was observed. Continued efforts to identify and design probes to target the abundant members of full scale AD systems [10,38,39] will allow comprehensive co-occurrence analyses to identify or confirm important metabolic dependencies of these groups.

Several of the most abundant genus-level-taxa in both mesophilic and thermophilic systems are also abundant in the activated sludge systems fed into these AD reactors (Fig 1B and 1C). These include ‘*Ca*. Promineofilum’ (Eikelboom 0092 morphotype: [14,40]), ‘*Ca*. Defluviifilum’ (Eikelboom 0803 morphotype: [13]), ‘*Ca*. Sarcinithrix’ (Eikelboom morphotype 0914: [41]), and ‘*Ca*. Amarolinea’ (Eikelboom 0092-like morphotype [35]), which all possess a filamentous morphology and can potentially contribute to bulking in activated sludge systems. Along with ‘*Ca*. Villigracilis’, these organisms represent the most abundant Chloroflexi genera in full-scale activated sludge systems with FISH probes already available to cover all of these groups [35]. Comparison of the abundances of these phylotypes across AS treatment plants and their corresponding AD systems shows a consistent reduction in their populations (Fig 1B and 1C). These observations would suggest they are being outcompeted by more specialized AD organisms, but gives no insight into potential activity of their cells—with high detected DNA sequencing-based AD abundances (up to 12%) leaving the possibility that they could still be numerically important active members of these AD systems.

The fate of immigrating species was therefore investigated in more detail at 5 WWTPs by comparing amplicon sequencing abundances with qFISH-based analyses, which require intact cells. The samples were selected based on the high abundance of Chloroflexi in the AD systems and included both mesophilic (3) and thermophilic (2) ADs. ‘*Ca*. Amarolinea’ was the most abundant AS phylotype in these plants, constituting up to 16% of the biomass in the AS communities (Table 2). Quantitative FISH analyses confirmed probable die-off of this genus in all plants tested. Notably, the population decreased from 16% of the biovolume to < 1% in the recipient AD system, despite being estimated at 8% of the AD community by amplicon sequencing (Table 2). ‘*Ca*. Villigracilis’ was also abundant in Ejby-Mølle, Aalborg East and Avedøre AS WWTPs at up to 3.3% of the biovolume. Interestingly, unlike any of the other AS abundant phylotypes considered, this genus was barely detected in any of the AD systems with either qFISH or DNA-based methods. This likely illustrates the difference in cellular envelope composition for the different phylotypes and the subsequent time taken to breakdown their cells, with the ‘*Ca*. Villigracilis’ being degraded relatively faster than the other influent groups assessed. Members of the ‘*Ca*. Promineofilum’, ‘*Ca*. Defluviifilum’ and ‘*Ca*. Sarcinithrix’ were below the reliable detection limit for qFISH in these sample sets. However, subjectively, there was a marked visible reduction in the number and fluorescent signal of these cells in AD samples relative to AS. From these preliminary observations, despite a high relative abundance observed with the commonly applied DNA-based methods, Chloroflexi abundant in AS systems generally appear to die-off when the biomass is fed into the ADs. This suggests that they are not active members of the AD community and that they are unlikely to make a substantial contribution to the transformations that underpin the function of these systems. Despite the known ability of some of these to ferment substrates under anaerobic conditions in AS, their apparent inability to survive in AD is likely due to the substantial differences in conditions between the AD and AS systems, including higher temperatures, higher salinity and higher ammonium concentration. Typical ammonium concentrations measured in AS are below 1–2 mgN/L, compared to the 1-3g/L found in the AD systems investigated in this study [4]. A similar increase is observed for the operational temperatures, that vary from 7–20°C in the AS investigated to 35–40°C and approx. 55°C for mesophilic and thermophilic ADs, respectively.

**Table 2:** Comparison of amplicon and qFISH relative abundances of Chloroflexi in the AS systems and their respective AD systems.

WWTP	Samples date	Abundance (%)*			
		Sequencing		qFISH	
		AS	AD	AS	AD
**“*Ca*. Amarolinea”**					
Aalborg West (T)	May 2014	11.9	6.7	13 ± 5	2.6 ± 1.5
Bjergmarken (T)	Oct. 2015	6.7	8	16 ± 5.2	< 1
Ejby-Mølle (M)	Oct.2015	3.1	1.3	3.6 ± 1.2	< 1
Aalborg East (M)	Aug. 2013	0.4	0.2	< 1	< 1
Avedøre (M)	Sep. 2015	0.4	0.2	<1	< 1
**“*Ca*. Villigracilis”**					
Aalborg West (T)	May 2014	n.d.	n.d.	n.d.	n.d.
Bjergmarken (T)	Oct. 2015	0.7	n.d.	< 1	n.d.
Ejby-Mølle (M)	Oct. 2015	3	n.d.	4 ± 1.9	n.d.
Aalborg East (M)	Aug. 2013	2.6	0.1	1.8 ± 1.1	n.d.
Avedøre (M)	Sep. 2015	3.1	n.d.	3.3 ± 1.2	n.d.
**“*Ca*. Promineofilum”**					
Aalborg West (T)	May 2014	1.6	1.6	< 1	< 1
Bjergmarken (T)	Oct. 2015	0.3	0.3	< 1	< 1
Ejby-Mølle (M)	Oct. 2015	1.2	0.8	< 1	< 1
Aalborg East (M)	Aug. 2013	0.7	1.3	< 1	< 1
Avedøre (M)	Sep. 2015	0.7	0.9	<1	< 1
**“*Ca*. Defluviifilum”**					
Aalborg West (T)	May 2014	1.2	0.7	< 1	< 1
Bjergmarken (T)	Oct. 2015	0.6	0.4	< 1	< 1
Ejby-Mølle (M)	Oct. 2015	1	0.3	< 1	< 1
Aalborg East (M)	Aug. 2013	1.7	1.7	< 1	< 1
Avedøre (M)	Sep. 2015	0.4	0.2	<1	< 1
**“*Ca*. Sarcinithrix”**					
Aalborg West (T)	May 2014	0.3	0.2	< 1	< 1
Bjergmarken (T)	Oct. 2015	0.4	0.3	< 1	< 1
Ejby-Mølle (M)	Oct. 2015	0.4	0.1	< 1	n.d.
Aalborg East (M)	Aug. 2013	0.7	0.6	< 1	< 1
Avedøre (M)	Sep. 2015	0.1	0.1	n.d.	n.d.

FISH probes applied to cover each group included: CFX64 (“*Ca*. Amarolinea”) [35], CFX763A+B (“*Ca*. Villigracilis”) [35], CFX197 (“*Ca*. Promineofilum”) [40], T0803-0654 (“*Ca*. Defluviifilum”) [13] and CFX1151 (“*Ca*. Sarcinithrix”) [35]. (T) Thermophilic (Aalborg West and Bjergmarken) and (M) mesophilic (Ejby-Mølle, Aalborg East and Avedøre) ADs; n.d. = positive cells not detected. Note that amplicon abundance values are relative for all bacteria whereas FISH biovolume estimates are relative to all microbial cells (incl. Archaea), which may partly account for lower values for the latter. Archaeal cells represent approx. 2–5% of the total biovolume by FISH in full-scale Danish AD systems [10].

## Conclusions

This study provides an insight into the structure of Chloroflexi populations in full-scale ADs. Application of qFISH has shown that abundant activated sludge populations appear to die-off when fed into AD systems despite being facultative anaerobes. As such, their numerical importance to AD systems appears to be overestimated by DNA-based methods. The genus-level-phylotypes described in this study are the abundant and active members of the Chloroflexi in AD systems; all having a thin, filamentous morphology that may indicate their potential importance to floc structure maintenance. Their confirmed relative high abundances indicates likely importance to the AD system function. Preliminary evidence suggests members of the Chloroflexi phylum abundant in AD may be primary fermenters, although the co-existence of different genera supports occupation of specific niches. Further characterisation of these abundant phylotypes will give an insight into the ecology and role of the phylum in ADs. Retrieval of high-quality genomes should be of high-priority for a more detailed insight into their metabolic potential. The FISH probes designed in this study provide a means of future *in situ* characterisation.
